# Does Voluntary Family Planning Contribute to Food Security? Evidence from Ethiopia

**DOI:** 10.3390/nu15051081

**Published:** 2023-02-21

**Authors:** Geteneh Moges Assefa, Muluken Dessalegn Muluneh, Sentayehu Tsegaye, Sintayehu Abebe, Misrak Makonnen, Woldu Kidane, Kasahun Negash, Abebaye Getaneh, Virginia Stulz

**Affiliations:** 1 Amref Health Africa in Ethiopia, Addis Ababa bole subcity, Woreda, Addis Ababa P.O. Box 20855, Ethiopia; 2 School of Nursing and Midwifery, Western Sydney University, Sydney, NSW 2751, Australia

**Keywords:** family planning, food security, voluntary family planning user, women, hierarchical logistic regression

## Abstract

This study aims to explore the effects of voluntary family planning (FP) utilization on food security in selected districts of Ethiopia. Quantitative research methods were used to conduct a community-based study among a sample of 737 women of reproductive age. The data were analyzed using a hierarchical logistic regression constructed in three models. The findings showed 579 (78.2%) were using FP at the time of the survey. According to the household-level food insecurity access scale, 55.2% of households experienced food insecurity. The likelihood of food security was lower by 64% for women who used FP for less than 21 months (AOR = 0.64: 95%CI: 0.42–0.99) in comparison to mothers who used FP for more than 21 months. Households having positive adaptive behaviors were three times more likely (AOR = 3.60: 95%CI 2.07–6.26) to have food security in comparison to those not having positive adaptive behaviors. This study also revealed that almost half of the mothers (AOR: 0.51: 95%CI: 0.33–0.80) who reported being influenced by other family members to use FP had food security, in comparison to their counterparts. Age, duration of FP use, positive adaptive behaviors, and influence by significant others were found to be independent predictors of food security in the study areas. Culturally sensitive strategies need to be considered to expand awareness and dispel misconceptions that lead to hesitancy around FP utilization. Design strategies should take into account households’ resilience in adaptive skills during shocks, natural disasters, or pandemics which will be invaluable for food security.

## 1. Introduction

According to the 1996 World Food Summit definition, food security exists when all people, at all times, have physical and economic access to sufficient, safe, and nutritious food to meet their dietary needs and food preferences for an active and healthy life [1,2,3,4]. Food security is comprised of four distinct pillars: food availability, food access, food utilization, and food stability (implies that access is not compromised by fluctuations in weather and market prices, by seasonality, and by economic or political shocks). Food security can be studied at the individual, household, national, and global level and be determined by the vulnerability and resilience of people [5,6,7,8].

Food security is a critical part of the Sustainable Development Goals (SDGs) and government policies in sub-Saharan Africa (SSA) and was initially focused on the production and availability of sufficient food. However, more importantly, these problems arose from government failure to integrate and keep track of rural food production, storage, and distribution systems [9,10,11,12].

Food insecurity is common in less-developed countries, including Ethiopia [13,14]. Food insecure households tend to have less adaptive capacity to maintain food security in cases of shocks, crises, natural disasters, or pandemics). These households have fewer resources to respond to new circumstances quickly and effectively, and limited ability to reduce vulnerability and the risk of impacts to human health including challenging family survival [15,16]. Studies suggest that rural households can work towards becoming food secure and sufficient by engaging more with FP [17,18].

FP enables individuals and couples to anticipate and attain their desired number of children, spacing, and timing of births [18,19]. A woman’s ability to space and limit pregnancies has a direct impact on her health and well-being as well as the outcome of each pregnancy [20,21]. Women who avoid unintended pregnancy and its consequences are more likely to participate in increasing their livelihood, thus increasing the socioeconomic power of their household [22,23,24].

Women entering the labor workforce supports increased resilience as women learn new ways to adapt their livelihood and reduce their health risks [25,26,27]. FP is associated with improved maternal and child health outcomes. Thus, having healthier children leads to fewer care burdens placed on mothers which frees them up to enjoy more leisure time while increasing their own adaptive capacity and reducing potential health and economic risks [28,29,30]. Persistent gender inequality existing in the community [27,30] means that women are less likely than men to be resilient in the face of external changes and shocks. This is often compounded by low rates of FP, early childbearing, and higher fertility rates. This leads to an early departure from school and lower participation in the labor workforce, making women less able to adapt to external shocks [31,32]. Food insecurity and malnutrition in adolescents and pregnant women are further compounded by gender discrimination, leading to an intergenerational cycle of nutrition problems [30,33,34]. This intergenerational cycle of nutrition problems manifests in stillbirths, miscarriages, low birth weight, growth failure, increased risk of maternal and neonatal mortality, and impaired cognitive development. Reduced nutrition also results in sub-optimal productivity in adults and reduced economic growth for the nation and at the local level [35,36,37,38]. For instance, a study conducted in Ethiopia showed the annual costs associated with child undernutrition are estimated at US$ 4.7 billion which is equivalent to 16.5% of the gross domestic product (GDP which is the standard measure of the value added created through the production of goods and services in a country during a certain period) [39]. 

Expanding access and use of voluntary FP has a positive impact on the food security of countries. Research findings suggest integrated programs are needed to utilize voluntary FP platforms as an entry point for food security [18,40]. Previous research on food security has shown that voluntary family planning can also decrease fertility rates and slow the pace of population growth, thus reducing food needs [41]. Vulnerability to food insecurity has a definite effect on the health of women. Programs should consider policy dialogue to encourage the integration of family planning, food security policies, strategies, action plans, and programs throughout the world, particularly in Asia and Africa [42]. Findings also show that socio-demographic data and positive adaptive behaviors can affect food security, and there is a need to mobilize political commitment and resources [43]. Research in Ethiopia demonstrates that slower population growth is achievable by addressing women’s existing needs for FP; thereby easing demand on food availability and altering population composition in ways that can enable food security. The conditions facing Ethiopia are shared by many countries throughout the developing world. Decision-makers, planners, and funders should consider FP as a potential food security strategy [44,45].

The Sustainable Development Goals (SDGs) make specific references to FP in regard to health and well-being, gender equality, and women’s empowerment. Ensuring all women and adolescent girls have access to high-quality, rights-based FP services contributes towards achieving these SDGs. The transformational benefits that FP brings to women, families, communities, and countries align with the five SDG themes of People, Planet, Prosperity, Peace, and Partnership [18]. Without universal access to FP and reproductive health services, the impact and effectiveness of other health and development interventions will be limited, will cost more, and will take longer to achieve [18,46]. Additionally, FP has been identified as a feasible solution to rapid population growth and its associated negative consequences [10,46,47]. Despite massive investments by governmental and non-governmental organizations, utilization and continuation rates of modern FP, especially in developing countries, remain low [32,33,48].

Evidence has shown that FP will contribute to stabilizing the fast-growing population [15,35,36,48] and will, in turn, support integration with food security and nutrition-focused programs [49]. However, there is a paucity of literature on the relationships between FP and food security. Therefore, this study aims to provide evidence on the association between food security and FP utilization and make recommendations to improve the integration of services in the regions.

## 2. Materials and Methods

### 2.1. Setting, Study Design, and Population

In Ethiopia, the lowland areas of Oromia and the Southern Nations, Nationalities, and Peoples’ Region (SNNPR) are among the most drought and famine-prone areas due to mixed farming production systems in the areas. Mixed farming is a farming method in which farmers grow crops and raise livestock on the same piece of land, and livestock manure is used to fertilize crop farmlands while the animals provide traction for farming. Most of the land resources (mainly the soil and vegetation) in the region have been highly degraded because of the interplay between environmental and human factors. These factors include climate change, population pressure, over-cultivation of the land, deforestation of vegetation and overgrazing, and high vulnerability to drought [36,50]. The study areas included the South Omo and the Wolaita zones of the SNNPR and the Bale zones in the Oromia region. According to the Central Statistical Agency population projection of Ethiopia, 378,993, 1,040,710, and 954,919 women are living in South Omo, the Wolaita zones of the SNNPR, and the Bale zones in the Oromia region, respectively [50], and the majority of the population is dependent on farming as a means of livelihood whilst other parts of the population are pastoralist. Nonetheless, recent evidence showed that 71.6% of households in the studied zones reported being food insecure [31,32]. The South Omo zone has the lowest number of rural households on average (0.4 hectares of land) in comparison to the national average of 1.01 hectares and an average of 0.89 hectares for the SNNPR. About 11.5% of the population is in a non-farm related job, in comparison to the national average of 25% and a regional average of 32% [35,51].

The contraceptive prevalence rate (CPR) among currently married women aged 15–49 in Ethiopia is 41%. Many currently married women use a modern method (41%), such as contraceptives, while only 1% use a traditional method such as periodic abstinence and withdrawal.

Urban women (48%) are more likely than rural women (38%) to use modern methods while the SNNPR’s usage was 44.5%. Use of contraceptives remained very low in Ethiopia and age, education level, number of children, wealth index, and marital status were factors that determined contraceptive use [52,53].

This study was conducted in 2020 and included mothers who had been using FP and non-FP users. Mothers who were using FP and have used FP were identified through the health posts’ Health Management Information System (HMIS). In Ethiopia, HMIS was launched in 2008 with the goal of creating a comprehensive and standardized national HMIS for evidence-based decision-making at all levels. It focuses on information use, data quality, human resources, and information communication to improve the health system’s efficiency and effectiveness. The HMIS and the Antenatal care (ANC) registry were used as a starting point to reach the study of women in the community. Selected kebeles’ health posts were used as a sampling frame to identify FP users and then employ a systematic random sampling method to select the final study participants. Once the women were identified in the HMIS and the ANC registry, health extension workers assisted in recruiting the women. This study employed a mixed quantitative and qualitative study design to explore how FP use contributed to household food security.

### 2.2. Sampling Procedures, Tools, and Data Collection

A total of 737 mothers who were both users and non-users of FP were included in the three zones for this study. The total sample size (737) was distributed across regions based on the probability proportional to size (PPS) method: 387 (52.5%), 138 (18.7%), 212 (28.8%) from Wolaita, South Omo, and Bale, respectively. The household questionnaire was developed by reviewing various literature including tools from the United States Agency for International Development (USAID), the Food and Agriculture Organization of the United Nations (FAO), and the World Food Program (WFP) [32,34]. Data were collected electronically using the mobile application ODK/KOBO in which the structured questionnaire with pre-coded answers was uploaded.

### 2.3. Data Analysis

Quantitative data collected were cleaned and analyzed using SPSS Version 28. Binary logistic regression was performed and variables having a *p*-value of <0.25 in bivariate analyses were included in the multivariable logistic regression model to test the existence of significant associations and to estimate respective odds ratios using 95% confidence intervals.

The status of food security in the study area was derived as a composite score using FAO food security indicators: food availability, food access, food utilization, and food stability at individual and household levels. Accordingly, this study used the FAO and USAID household-level food insecurity access scale (HFIAS) for the measurement of food insecurity. Respondents were asked about occurrence questions with a recall period of four weeks (30 days). The respondent was first asked an occurrence question—that is, whether the condition in the question happened at all (yes or no). If the respondent answered “yes” to an occurrence question, a frequency-of-occurrence question was asked to determine whether the condition happened rarely (once or twice), sometimes (three to ten times), or often (more than ten times) in the past four weeks. The questionnaire consisted of nine occurrence questions that represented a generally increasing level of severity of food insecurity (access) and nine “frequency-of-occurrence” questions that were asked as a follow-up to each occurrence question to determine how often the condition occurred. The frequency-of-occurrence question was skipped if the respondent reported that the condition described in the corresponding occurrence question was not experienced in the previous four weeks (30 days) [54]. 

Household’s HFIAS categories: 1 = Food Secure, 2 = Mildly Food Insecure, 3 = Moderately Food Insecure, 4 = Severely Food Insecure.

A brief questionnaire was used to ask respondents about the frequency of their household’s consumption of eight different food groups over the previous seven days. The HFIAS for Measurement of Food Access was used to enquire about information over the previous four weeks and food availability in the households.

The Food Consumption Score (FCS) was calculated and the consumption frequencies were summed and multiplied by the standardized food’s group weight. Households can then be further classified as having “poor (0–21)”, “borderline (21.5–35)”, or “acceptable (>35)” food consumption by applying the WFP’s recommended cut-offs to the food consumption score. Food security status was also measured by applying the HFIAS. Hierarchical logistic regression was conducted to identify factors affecting food security at different levels, including individual and community-level factors. Accordingly, model 1 included individual-level factors (family planning utilization, socio-demographic data) and model 2 included community-level factors (service accessibility, support to use FP, decision-making, and influencers). Significant variables from model 1 and model 2 were included in the final model and 3 for further analysis to identify factors affecting food security in the study area. Additionally, some of the variables were categorized and operationally defined as follows for analysis purposes: 

For this study, food security was dichotomized as food secure and insecure households, with insecure including mild, moderate, and severe food insecurity. Positive adaptive behaviors refer to the potential to maintain food security in cases of shocks, crises, natural disasters, or pandemics. This study measured self-reported ratings by participants. Food stability implied that access to food was not compromised by fluctuations in weather and market prices, by seasonality, and by economic or political shocks. Influence by significant others refers to any person or persons with a strong influence on an individual’s self-concept in family planning utilization (for example, boyfriend/girlfriend, spouse, mother-in-law and partner).

### 2.4. Ethical Considerations

A letter of permission to conduct this study was obtained from the Wolaita, Bale, and South Omo zones and each district office in these zones. Study participants were assigned unique ID numbers, and names and any other identifiers did not appear on any of the principal data collection instruments. All study materials were secured, with access only granted to research team members for data management and analysis. Informed consent was obtained from every participant before proceeding with interviews. Each study subject who agreed to participate was interviewed in a private location, thus, each participant’s privacy was protected. Additionally, households that showed poor food security were linked to support programs. 

## 3. Results

### 3.1. Socio-Demographic Characteristics

A total of 737 women were included in the three zones and the mean age of the study participants was 28.9 years old (SD ± 6.6), including pregnant women. Almost all women (96.9%) who participated in this study were residing in a rural area at the time of data collection and 703 (95.4%) were married. More than half of the women (55.2%) were illiterate and 254 (34.5%) had attended primary school. Their husbands’ literacy and educational status were similar; 330 (44.8%) were illiterate and 277 (37.6%) had attended primary school. The main sources of income for their families were land cultivation, (n = 271, 36.8%) and animal husbandry (n = 270, 36.6%).

At the time of the survey, more than three-quarters of mothers (n = 566, 76.8%) were not working, and 158 (21.4%) of mothers ran their own businesses. 

FP was defined for the study participants before the interviews. The study participants were asked about their source of FP information. Nearly all participants (96.3%) reported health extension workers as their main source of FP information and 706 (95.7%) women responded that health extension workers are their most trusted source of information for FP (see Table 1). 

### 3.2. Access and Use of FP

The mean family size among study participants was 5.8 (SD ± 1.9). The majority of mothers, 676 (91.7%), reported that they had ‘visited a health facility/a doctor to receive FP information/services’. Six hundred and forty-three (87.2%) participants identified health posts as the closest health facility that they normally accessed for health services. The nearby health facility for most of the mothers was located within ten km or less from their homes. At the time of data collection, 66 (9.0%) were pregnant. About 706 (95.8%) mothers heard or knew of FP services and 236 (32%) mothers were able to list three or more of the modern FP methods. 

**Figure 1:**
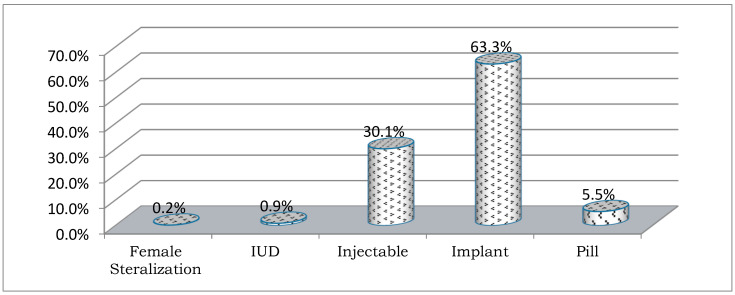
Modern Family Planning Utilization Method, December 2020.

A large proportion of mothers 642(87.1%) supported the use of FP to delay or prevent pregnancy. About 600 (81.4%) mothers have been using FP services during the course of their fecund lifetime and 579 (78.2%) were using FP at the time of the survey. Among current users of FP 579(78.2%), the mean duration of mothers using FP was 21.2 months. This shows the overall type of FP utilization method. Figure 1 shows nearly two-thirds of current users 366 (63.3%) were using implants, followed by an injectable form of contraception (174, 30.1%). Regarding the availability of FP commodities, 302 (41%) women reported that commodities were available when they visited health facilities.

When asked about the reason for using FP (among 600 FP users), 165 (27.5%) mothers responded by stating spacing or limiting family size, 301 (50.2%) for delaying pregnancy, while 273 (45.5%) responded that it provided enough time to provide attention for family, children, and personal advancement. Among non-users, 34 (24.8%) women did not use FP as they feared the side effects and 15 (10.9%) of them did not use FP due to religious reasons or because their husband/family did not allow them to use FP. 

The decision-making process to use FP was also influenced by local cultural views and customs, which encouraged them to have many children. Moreover, a significant proportion of women 212 (28.8%) worried about the side effects of FP. The findings of this study suggest a significant proportion (236–32%) of mothers seek input from their husbands or a close relative, meaning that relatives contributed to decision-making about FP methods as well. A negligible number 22 (2.9%) of women make their own decisions about their reproductive health service utilization. Overall, 327 women (44.3%) responded that their choice to use FP had been influenced by someone else.

### 3.3. Status of Food Access, Security, Consumption, and Availability

#### 3.3.1. Food Access

The findings of this study show that the frequency and variety of food available were major challenges faced by the study population. In Table 2, the occurrence questions show an increasing level of food insecurity that are asked as a follow-up to each occurrence question to determine how often the condition occurred among FP users and non-users (*p*-values also show the associations). Sixty percent of households (444) reported that in the past four weeks, they worried that their household would not have enough food to eat. Sixteen percent of households (119) reported that in the past four weeks, they faced days with no food to eat in their household because of a lack of resources. Additionally, 64 (8.7%) households reported there were days when the mother, or any household member, went to sleep hungry because there was not enough food, while 27 (3.7%) households experienced a mother, or any household member, going the whole day and night without eating because there was not enough food. This indicates that a significant portion of households were considered severely food insecure. The household level for food access was also disaggregated by FP users and non-users, showing that households with FP users were more likely to be able to access food in comparison to non-users (see Table 2). 

Shows food access and security status among women based on HFIAS. As shown in the Table 3, only 44.8% of the surveyed households had food secured. 

#### 3.3.2. Food Security

**Figure 2:**
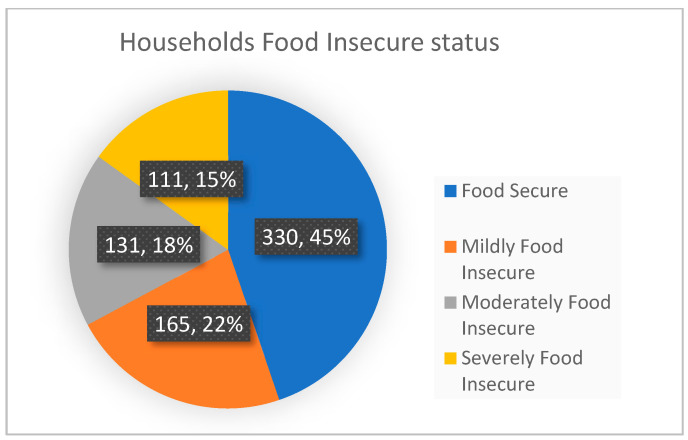
Status of food security, Ethiopia, December 2020.

This study’s findings revealed that 45% of the study households were food secure and 22%, 18%, and 15% of the households were classified as experiencing mild, moderate, and severe food insecurity. This study also found that food security status differed with FP users and family size. Women who were using FP were more secure 326 (46.2%) than their counterparts (n = 68, 9.6%). On the other hand, households who had a family size of six or less were more food secure (n = 224, 30.4%) than households with a family size greater than six (n = 183, 24.8%). (see Figure 2 and Table 4).

**Figure 3:**
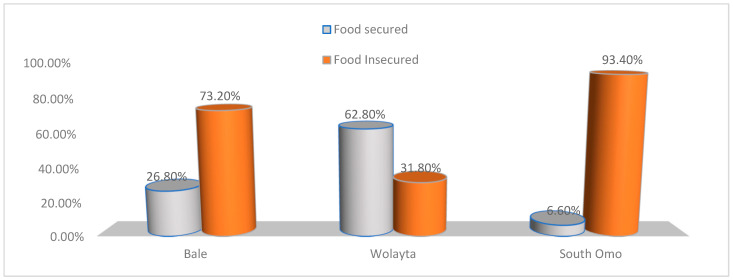
Status of Food Security by Cluster, Ethiopia, December 2020.

Food security status varied across different study clusters; South Omo was the least food secure (6.6%) cluster followed by the Bale cluster (26.8%) (Pearson chi-square = 195, *p* < 0.0001). The Wolaita zone had the highest food security in comparison to other zones (68.2%) (see Figure 3 ). 

**Figure 4:**
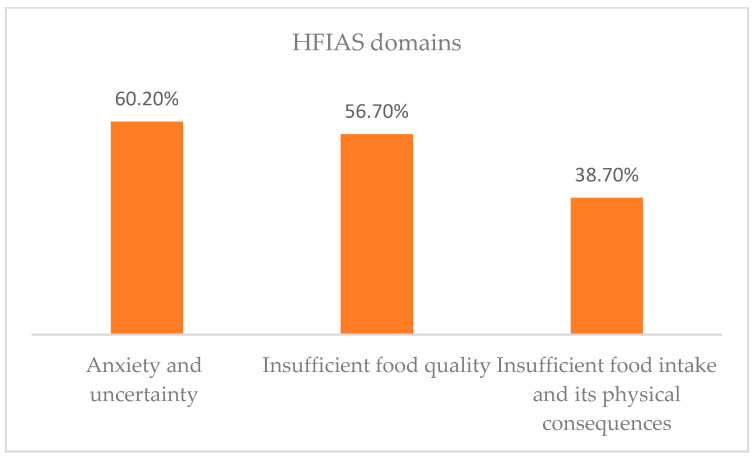
Status of household food insecurity access domains, Ethiopia, December 2020.

The nine questions were also grouped into domains based on their characteristics. Sixty point two percent (444) of households were classified in the anxiety and uncertainty domain, 56.7% (418) were classified into the insufficient food quality domain, and 38.7% (285) were classified into the insufficient food intake and its physical consequences domain (see Figure 4).

#### 3.3.3. Food Consumption

Food consumption scores were calculated by following the World Food Program’s (WFP) recommendation. Food consumption scores were calculated as a composite score based on dietary diversity, food frequency, and relative nutritional importance of different food groups at the household level. The food consumption score per week represents the median of each food category. Based on the formula, for example, Food Consumption score (FCS) = Average consumption for each food group*weight for each group. Based on this, the average food consumption for the study area was 37.5. 

According to the WFP recommendation, this result is categorized within the ‘Acceptable’ food consumption range. The food consumption score was also disaggregated by zones and is shown in Table 5. The Bale and South Omo zones had an acceptable food consumption score while the Wolaita zone scored ‘borderline’ for food consumption.

#### 3.3.4. Food Availability 

The study measured food availability based on the average production, storage, sale, and purchase per household. Accordingly, 55.0% and 52.8% of women had good production and storage levels in the study area (South Omo, the Wolaita zones of the SNNPR, and Bale in the Oromia region) in the past year, respectively. In contrast, 75.6% of mothers had less than the average sale capacity, and 46.0% achieved below the study population’s average annual purchase rate.

In the cases of shocks, crises, natural disasters, and pandemics (such as COVID-19), 6.2%, 22.0%, 46.3%, and 25.5% of the participants rated (their own rating) that they had excellent, very good, good, and poor household positive adaptive behaviors, respectively. 

### 3.4. Association between FP and Food Security

The study used hierarchical logistic regression to assess individual (Level 1) and community-level (Level 2) factors that influenced the food security level of households. The “15–19 years” age group was used as the reference group because the age category was based on studies and current CPR in Ethiopia, and this helped to interpret our study’s hypotheses.

Variables having *p* < 0.25 in bivariate analyses were included in a multivariate analysis in model III. Model III results in bold print represent associations that were found to be statistically significant (*p*-value < 0.05).

Multicollinearity was tested using the variance inflation factor (VIF) before the analysis model. Accordingly, age, duration of FP use, good adaptive behavior, and influence by significant others were found to be independent predictors of food security in the study areas. The final model showed that the likelihood of food security was lower by 82% and 92% for women aged 20–29 years and 30–49 years, respectively. The likelihood of food security was lower by 64% for women who used FP for less than 21 months in comparison to mothers who used FP for more than 21 months. Households having positive adaptive behaviors were three times more likely to have food security in comparison to those not having positive adaptive behaviors. The study also revealed that almost half of the mothers who reported being influenced by other family members to use FP were food insecure, in comparison to their counterparts (see Table 6). 

## 4. Discussion

This study has examined the effect of FP on food security in Wolaita, South Omo, and the Bale zones of Ethiopia. This study was based on a representative sample of 737 women drawn randomly from selected households. It is understood from the analysis that only 44.8% of women (95%CI: 1.56–1.59) had food security. This finding is lower than a study conducted in Nigeria (44.8% vs. 58.1%) and higher than studies in Ethiopia (44.8% vs. 28.4%). The differences in the reported figures could arise from differences in the study setup (such as socio-demographic and cultural differences) or variations in scope (such as sample size) or both [4,10,12,39,42,45,49]. 

The study findings also revealed that longer periods of FP utilization contributed to food security. This finding concurs with the recommendations of integrating FP in agriculture and education to achieve the SDGs and Universal Health Coverage (UHC) by 2030 [8,9,13,15,16,18,43,45,46,49]. This study also showed that 63.3% of women used implants, which could be attributed to two major reasons. Firstly, the majority of the mothers lived in pastoralist communities and they moved from place to place for longer periods, which meant that they preferred longer-term FP methods. Secondly, the ministry of Ethiopia and the local government had worked to scale up access to services, and the closest health facilities, including health posts, were able to provide implants for women. Deep-rooted cultures affect women’s decision-making processes and they are greatly influenced by cultural norms and family members. 

The findings reported in this research also showed that an increase in age is inversely associated with food security. This finding is also consistent with other similar studies conducted around the world, including Ethiopia, which concurred with these findings. This finding suggests that as age increases the number of children/family also increases and an increase in family number might contribute to food insecurity [37,40,42,49].

As per the self-reported ratings, households having positive adaptive behaviors (in cases of shocks, crises, natural disasters, or pandemics) had good food security. This finding concurred with similar studies [7,9,10,21,38,43,44].

This study also revealed that almost half of the mothers (AOR: 0.51: 95%CI: 0.33–0.80) who reported being influenced by other family members to use FP had food insecurity, in comparison to their counterparts. Given the fact that most women in Ethiopia are less educated and are living in a patriarchal society, their autonomy and participation in decision-making about their own health could be limited. This in turn limits the possibilities of open discussion with their husband, health workers, and other members of the community [15,16,17,36,39,43,46,47,50]. The implications of this finding could be extended to reaching the mothers and those who influence her decisions with intensive health education.

Investing in FP is a development ‘‘best buy’’ that can accelerate achievement across the five SDG themes of People, Planet, Prosperity, Peace, and Partnership, which, in turn, accelerates the achievement of the SDG 2 ‘No hunger’ [4,9,16,44,46,48].

## 5. Limitations

Since this study was conducted in an agricultural production time, seasonal variation may affect the findings. One important commonality is the seasonality of food shortages, although it is more pronounced in rural areas. The months of June, July, August, and September are identified as food production shortages or deficient periods for many households in the study zones. Variables having less significance (*p* < 0.25) in the bivariate analyses included the multivariate analyses that still contribute substantially. Further research is recommended by extending the duration of FP in future studies with matching methods to control for confounding factors.

## 6. Conclusions and Policy Implications

FP is a globally and nationally recommended strategy to ensure the food security of a nation and facilitate economic development. FP brings transformational benefits to women, families, communities, and countries. This study found that the longer the duration of FP utilization, the more likely that households had better food security.

Age, duration of FP use, positive adaptive behaviors, and influences by others in the processes of using FP were found to be independent predictors of household-level food security. This study helps to refocus the lens on integrating FP utilization as part of a food security strategy. Older age, less positive adaptive behaviors, and households that were influenced by significant others also experienced food insecurity. Culturally sensitive strategies need to be considered in order to expand awareness and dispel misconceptions that lead to hesitancy around FP utilization. It is also recommended that sectors integrate and expand FP services as part of food security programs for women to easily access these services. Empowerment of women in decision-making should be addressed by multi-sectoral approaches and community advocacy campaigns for FP utilization. Increasing design strategies for households to be resilient and improve their adaptive skills during shocks, natural disasters, or pandemics is invaluable for food security. 

The findings reported in this study provide vital evidence to inform policy and decision-makers to respond to food security and FP utilization in alignment with the SDGs’ target by 2030. Many community members still do not have a deep understanding of the benefits of FP utilization and how it can contribute to food security. Health extension workers were the main source of information for FP and this helps to easily adapt to community demands. The program is also dynamic enough to shift tasks between health centers and the community. Health extension programs are suggested as an interim strategy for reaching community FP utilization.

Cultural ideas, norms, and customs are deeply embedded in societal structures and religious institutions. Thus, community dialogues can help catalyze shifts in power so that women and girls have increased and equitable decision-making power about FP. Hence, there is a need to work on multi-sectoral approaches to ensure FP utilization is given the necessary attention at all levels.

## Figures and Tables

**Table 1:** Source of Information for Family Planning, Ethiopia, December 2020, (N = 737).

Sources of Information		Frequency	%
Family Planning Source of information	Television	5	0.7
	Health care providers	16	2.2
	Health Extension Workers	710	96.3
	No common source	6	0.8
Trusted source of information for Family Planning	Television	5	0.7
	Health care providers	18	2.5
	Health Extension Workers	706	95.7
	No common source	8	1.1

Key: No common source refers to those who did not have a common source to refer to as a source of information. Participants were asked to choose accessible information sources and then, from their lists, they were able to select their most trusted information sources.

**Table 2:** Household level food access disaggregated by FP User and Non-User, December 2020 (n = 737).

Variables (In the Past Four Weeks)		Overall		FP User	Non-User	*p*-Value
		Frequency	Percent	Percent	Percent	
Did you worry that your household would not have enough food? Were you or any household member not able to eat the kinds of foods you preferred because of a lack of resources?	Yes	444	60.2	62.0	53.8	0.062
	Yes	467	63.4	64.4	59.5	0.254
Did you or any household member have to eat a limited variety of foods due to a lack of resources?	Yes	382	51.8	52.0	51.3	0.872
Did you or any household member have to eat some foods that you really did not want to eat because of a lack of resources to obtain other types of food?	Yes	292	39.6	37.3	48.1	0.014
Did you or any household member have to eat a smaller meal than you felt you needed because there was not enough food?	Yes	299	40.6	39.0	46.2	0.104
Did you or any household member have to eat fewer meals in a day because there was not enough food?	Yes	203	27.5	25.0	36.7	0.004
Was there ever no food to eat of any kind in your household because of a lack of resources to get food?	Yes	119	16.1	14.7	21.5	0.038
Did you or any household member go to sleep at night hungry because there was not enough food?	Yes	64	8.7	8.3	10.1	0.467
Did you or any household member go a whole day and night without eating anything because there was not enough food?	Yes	27	3.7	3.6	3.8	0.919

**Table 3:** Frequency of Food Security Status, December 2020 (N = 737).

Variables		Frequency	%	How Often Did This Happen?		
				Rarely	Sometimes	Often
In the past four weeks, did you worry that your household would not have enough food?	Yes	444	60.2	44.8 ^1^	51.2 ^2^	4.0 ^2^
In the past four weeks, were you or any household member not able to eat the kinds of foods you preferred because of a lack of resources?	Yes	467	63.4	44.1 ^2^	54.6 ^2^	1.3 ^2^
In the past four weeks, did you or any household member have to eat a limited variety of foods due to a lack of resources?	Yes	382	51.8	64.6 ^2^	34.6 ^3^	0.8 ^3^
In the past four weeks, did you or any household member have to eat some foods that you really did not want to eat because of a lack of resources to obtain other types of food?	Yes	292	39.6	59.5 ^2^	38.4 ^3^	2.1 ^3^
In the past four weeks, did you or any household member have to eat a smaller meal than you felt you needed because there was not enough food?	Yes	299	40.6	72.9 ^3^	26.7 ^3^	0.4 ^4^
In the past four weeks, did you or any household member have to eat fewer meals in a day because there was not enough food?	Yes	203	27.5	86.2 ^3^	13.8 ^3^	0.0 ^4^
In the past four weeks, was there ever no food to eat of any kind in your household because of lack of resources to get food?	Yes	119	16.1	90.8 ^4^	9.2 ^4^	0.0 ^4^
In the past four weeks, did you or any household member go to sleep at night hungry because there was not enough food?	Yes	64	8.7	90.6 ^4^	9.4 ^4^	0.0 ^4^
In the past four weeks, did you or any household member go a whole day and night without eating anything because there was not enough food?	Yes	27	3.7	88.9 ^4^	11.1 ^4^	0.0 ^4^

Key: Interpretation of findings was made based on the household-level food insecurity access scale (HFIAS) and the superscript descriptions presented below. ^1^ Food Secure; ^2^ Mildly Food Insecure; ^3^ Moderately; ^4^ Severely Food Insecure.

**Table 4:** Comparison of Food Security Status, currently using Family Planning, and Family size, Ethiopia, December 2020 (N = 737).

Variable		Status of Food Security		*p*-Value
		Food Insecure	Food Secure	
Ever used family planning	Yes	274 (38.8%)	326 (46.2%)	0.071
Family size (mean used as cut-off)	≤6	133 (18.0%)	224 (30.4%)	0.000
	>6	197 (26.7%)	183 (24.8%)	
Currently using family planning	Yes	244 (33.1%)	335 (45.5%)	0.007

**Table 5:** Food Consumption measurement in Wolaita, South Omo, and Bale zones, Ethiopia, December 2020.

Types of Food	Weight	Average Food Consumption Per Week (in Days)			
		Average	Bale	Wolaita	South Omo
Main staples Pulses	2	6	7	5	4
	3	3	7	2	4
Vegetables and leaves	1	2	2	1	3
Fruits	1	1	0	1	1
Meat/Beef, goat, poultry, eggs, and fish	4	0	1	0	4
Milk yogurt and other diaries	4	3	7	1	7
Sugar and sugar products	0.5	1	7	0	1
Oils, fat, and butter	0.5	2	7	0	2
FCS rating		37.5	76	24	69.5
Category			Acceptable	Borderline food consumption	Acceptable

Key: Field note indicates that households chose to reduce the variety of food as a primary strategy to reduce the impacts of food insecurity in their family.

**Table 6:** Hierarchical logistic regression on food security, Ethiopia, December 2020.

Variables	Crude Odd Ratio (COR) (95% CI)	Adjusted Odd Ratio (AOR) (95% CI)		
		Model I	Model II	Model III
**Age**				
15–19	1	1		1
20–29	0.42 (0.21–0.85)	0.20 (0.05–0.89)		**0.18 (0.04–0.83)**
30–49	0.18 (0.09–0.36)	0.09 (0.02–0.39)		**0.08 (0.02–0.35)**
Family members				
≤ 6	1	1		
> 6	0.55 (0.41–0.74)	1.05 (0.64–1.72)		
Current working Status				
Working	2.19 (1.55–3.12)	0.59 (0.34–1.05)		
Not-working	1	1		
Currently using family planning				
Yes	1.61 (1.01–3.11)	2.01 (1.12–2.32)		1.17 (0.05–1.89)
No		1		
Family planning used				
≤21 months	0.43 (0.29–0.64)	0.53 (0.35–0.81)		**0.64 (0.42–0.99)**
>22 months	1	1		1
Adaptive Capacity (households’ own scoring)				
Excellent	2.82 (1.46–5.46)	3.08 (1.19–7.96)		2.13 (0.93–4.85)
Very good	1.58 (1.03–2.45)	2.00 (1.04–3.85)		1.56 (0.85–2.85)
Good	5.06 (3.45–7.43)	3.91 (2.25–6.79)		**3.60 (2.07–6.26)**
Poor	1	1		
Distance of health facility				
Less than or equal to 10 Kilometre	0.66 (0.39–1.12)		0.75 (0.43–1.32)	
Greater than 10 Kilometre	1		1	
Future support FP use				
Yes	0.74 (0.43–1.25)		0.77 (0.43–1.37)	
No	1		1	
Decision-maker for FP utilization				
Myself	1		1	
My husband	0.73 (0.53–1.00)		0.71 (0.50–1.00)	
Close relatives	0.78 (0.34–1.87)		0.72 (0.29–1.76)	
Influenced by significant others				
Yes	0.44 (0.32–0.59)		0.45 (0.33–0.62)	**0.51 (0.33–0.80)**
No	1		1	

Model III results in bold print represent associations that were found to be statistically significant (*p*-value < 0.05).

## Data Availability

The data presented in this study are available on request from a corresponding author. The data are not publicly available due to privacy reasons.
